# Bile Facilitates Human Norovirus Interactions with Diverse Histoblood Group Antigens, Compensating for Capsid Microvariation Observed in 2016–2017 GII.2 Strains

**DOI:** 10.3390/v12090989

**Published:** 2020-09-05

**Authors:** Michael L. Mallory, Lisa C. Lindesmith, Paul D. Brewer-Jensen, Rachel L. Graham, Ralph S. Baric

**Affiliations:** Department of Epidemiology, University of North Carolina, Chapel Hill, NC 27599, USA; mlmallor@live.unc.edu (M.L.M.); lisal@unc.edu (L.C.L.); pbj@email.unc.edu (P.D.B.-J.); rlgraham@ad.unc.edu (R.L.G.)

**Keywords:** norovirus, immunity, bile, blockade antibody, histo blood group antigen

## Abstract

Human norovirus (HuNoV) is the leading cause of global infectious acute gastroenteritis, causing ~20% of reported diarrheal episodes. Typically, GII.4 strains cause 50–70% of yearly outbreaks, and pandemic waves of disease approximately every 2–7 years due to rapid evolution. Importantly, GII.4 dominance is occasionally challenged by the sudden emergence of other GII strains, most recently by GII.2 strains which peaked in 2016–2017, dramatically increasing from 1% to 20% of total HuNoV outbreaks. To determine if viral capsid evolution may account for the sudden rise in GII.2 outbreaks, Virus Like Particles (VLPs) of two 2016–2017 GII.2 strains were compared by antigenic and histo blood group antigen (HBGA) binding profiles to the prototypic 1976 GII.2 Snow Mountain Virus (SMV) strain. Despite >50 years of GII.2 strain persistence in human populations, limited sequence diversity and antigenic differences were identified between strains. However, capsid microvariation did affect HBGA binding patterns, with contemporary strains demonstrating decreased avidity for type A saliva. Furthermore, bile salts increased GII.2 VLP avidity for HBGAs, but did not alter antigenicity. These data indicate that large changes in antigenicity or receptor binding are unlikely to explain GII.2 emergence, in contrast to the pandemic GII.4 strains, and indicate that host factors such as waning or remodeling of serum or mucosal immunity likely contributed to the surge in GII.2 prevalence.

## 1. Introduction

Human norovirus (HuNoV) is the leading cause of infectious acute gastroenteritis (AGE), with ~685 million cases globally occurring every year [1]. Of all reported diarrheal episodes, 20% are HuNoV related, primarily afflicting the young, elderly, and immunocompromised, with over 200,000 deaths per year largely resulting from complications from dehydration and malnutrition [2,3,4,5]. Extensive disease burden within these highly susceptible populations warrants development for an effective HuNoV vaccine, which is complicated by extensive antigenic diversity between HuNoV strains. Typically, 50–70% of yearly HuNoV outbreaks are caused by genotype GII.4 strains, which cause pandemic levels of disease approximately every 2–7 years due to rapid evolution and recombination [1,6,7]. However, GII.4 dominance is occasionally subverted by other GII strains. During the 2014–2015 HuNoV winter season, global outbreaks of GII.17 dramatically increased. Additionally, during the 2016–2017 HuNoV winter season, global outbreaks of GII.2 strains dramatically increased, from ~1% to 20% of total HuNoV outbreaks, and then quickly subsided the following season [8,9,10]. The mechanisms regulating the sudden GII strain emergence and subsequent disappearance is unknown, but represents a fundamental epidemiologic phenomenon in norovirus epidemiology. Consequently, the stark increase in GII.2 prevalence during the 2016–2017 season warranted exploration of antigenic changes within the viral capsid, along with viral/host interactions that could have enabled GII.2 to emerge, concepts essential for understanding norovirus immunity and susceptibility and, subsequently, vaccine design.

Belonging to the Caliciviridae family, noroviruses are ~7.5 kb single-stranded, positive-sense RNA viruses with 3 open reading frames (ORFs) [11]. ORF1 encodes the non-structural proteins, including the error-prone RNA dependent RNA polymerase (RdRP), while ORF2 and ORF3 encode the major capsid protein (viral protein 1, VP1), and the minor capsid protein (VP2), respectively [12]. VP1 is composed of the N-terminal shell (S) and C-terminal protruding (P) domains, which can be further broken down into P1 and P2 subdomains [13]. The P2 domain comprises the majority of antigenically distinguishable sites, or epitopes, on the different HuNoV genotypes, and is responsible for host cell attachment via Histo-Blood Group Antigens (HBGA) [14,15,16,17,18,19,20]. Transfection of the HuNoV VP1 gene into mammalian cells results in the expression of VP1 protein that self-assembles into Virus Like Particles (VLP) that are morphologically and antigenically indistinguishable from native virions, based on morphology, HBGA, and antibody recognition [21,22,23].

Currently, there are five classified genogroups of noroviruses (GI-GVII), with genogroups GI and GII causing the majority of human infections [13,24]. Within genogroups GI and GII, there are over 30 recognized genotypes. These genogroups share less than 50% sequence identity, with strains of the same genotype having less than 20% difference in homology [25,26]. The GII strains cause ~90% of yearly outbreaks, largely dominated by GII.4 strains [13,26]. Over the past three decades, sequential pandemic outbreaks of GII.4 strains have been linked to antigenic drift within GII.4 VP1 [17,21,27,28]. In-depth studies of the variation between pandemic GII.4 strains utilizing monoclonal antibodies have identified both conserved and strain-specific epitopes within the capsid, distinguishing key epitopes responsible for immune escape and new strain emergence [29,30].

For GII.2 strains, which typically cause few reported outbreaks, the conditions leading to emergence are not as clearly defined, as GII.2 capsid sequences circulating over the last ~50 years have shown limited variation [31]. It has been theorized that recombination events with the GII.16 RNA-dependent RNA Polymerase (GII.P16 RdRP) produced GII.P16_GII.2 strains with increased epidemic potential leading to the 2016–2017 outbreaks recorded in multiple countries, such as United States, France, Germany, Mainland China, Taiwan, Hong Kong, and Japan [9,32,33,34]. However, GII.2 strains with GII.P16 RdRP were previously detected in outbreaks occurring during the 2009–2010 norovirus season in Osaka, Japan, yet the GII.2_GII.16P viruses circulated at lower levels of detection in comparison to 2016–2017 [35]. Additionally, GII.P16 RdRP recombination has occurred with other capsid genotypes, such as GII.3 and GII.13, which did not show a dramatic increase in strain circulation [36]. GII.2 capsid strains have also circulated with other polymerases that did not lead to a significant increase in outbreaks [35]. These data are complicated by unknown effects of mutations in the GII.P16 polymerase [37]. Bioinformatics analyses indicate that residue changes within the capsid of 2016–2017 GII.2 strains may have resulted in antigenic changes [32]. However, these predictions have not been validated by antibody studies and are further complicated by the lack of verified GII.2 epitopes.

HBGA expression patterns determine host susceptibility to HuNoV infection in a strain specific manner [38]. GII.2 strains do not bind or bind weakly to many HBGAs in vitro but infect individuals of blood types A, B, O in vivo, indicating that factors beyond HBGAs are important in HuNoV infection [31,39,40]. Recent studies of GII.2 have demonstrated that bile acids improve HBGA ligand affinity [41]. Similarly, bile acids increase engagement of the P domain of mouse norovirus with the CD300lf receptor and enhance growth of some HuNoV strains in human intestinal enteroid cells [42,43]. These data suggest that intestinal co-factors support calicivirus receptor binding, including GII.2 -HBGA binding, and may also impact virus susceptibility [41].

In this study, we compared the evolution, antigenicity and carbohydrate binding patterns of emergent GII.2 outbreak strain VLPs to the prototype GII.2 1976 Snow Mountain Virus VLP to determine if capsid sequence changes identified in contemporary strains contributed to virus escape from herd immunity, or if the virus gained the ability to infect a broader population by expanding receptor ligand usage, as has been described for emergent GII.4 stains.

## 2. Materials and Methods

### 2.1. Production of Virus like Particles

VLPs utilized in this study were produced by synthesizing HuNoV ORF2 genes optimized for human expression (Bio Basic Inc., Markham, ON, Canada) with ApaI and AscI restriction sites for direct ligation into the pVR21 VEE replicon vector. After sequence-verification, pDNA was propagated in *Escherichia coli* Top 10 cells (Invitrogen, Waltham, MA, USA), purified utilizing a QIAprep Miniprep kit (Qiagen, Germantown, MD, USA), and linearized with NotI. After gel purification with the QIAquick Gel Extraction Kit (Qiagen, Germantown, MD, USA) and generating mRNA using mMESSAGE mMachine T7 transcription kit (Invitrogen, Waltham, MA, USA), mRNA was electroplated into BHK-21 cells (ATCC CCL-10, Manassas, VA, USA) for production of VLP [22,23]. After 27 h, VLPs were harvested from cells as described in [22]. VLP concentration and particle integrity were determined by BCA assay (Pierce/Thermo-Fisher, Waltham, MA, USA) and visualization by electron microscopy of ~40 nm particles. 

### 2.2. GII.2 Homology Model

The amino acid sequences of GII.2 1976 SMV (accession no. MT767436), GII.2 Chapel Hill (MT767367), and GII.2 Nashville (ARR95874) were aligned using Geneious Prime version 2019.2.1 (Geneious, San Diego, CA, USA). PDB accession no. 2OBT was used as a template for generation of P domain structural homology models utilizing PyMOL 2.2.0 (Schrodinger, New York City, NY, USA). For GII.2 CH alanine mutant VLPs, sequence changes observed between GII.2 SMV and contemporary GII.2 VLP were parsed into 5 groups and alanine mutations designed in the GII.2 Chapel Hill backbone. 

### 2.3. Enzyme-Linked Immunoabsorbent Assay (EIA)

EIAs were performed as described [44]. EIA plates were coated with VLP (0.25 g/mL) in PBS for 4 h and blocked over night at 4 °C in 5% non-fat dried milk in PBS-0.05% Tween-20 (blotto) before addition of decreasing two-fold serial dilutions of mouse mAb or rabbit pAb. All antibody incubations were conducted at 37 °C in blotto. Bound antibody was detected by either anti-mouse IgG-HRP or anti-rabbit IgG-HRP (GE Healthcare, Chicago, IL, USA), and color developed with 1-Step Ultra TMB ELISA HRP substrate solution (Thermo-Fisher, Waltham, MA, USA). Fifty percent effective concentration (EC_50_) was determined for antibodies with optical densities (ODs) ≥ 3× background at 2 µg/mL. Data were fit using sigmoidal dose–response analysis of nonlinear data in GraphPad Prism 8.3.0 (GraphPad Software, La Jolla, CA, USA). Monoclonal Abs below the limit of detection were assigned an EC_50_ of 2× the assay upper limit of detection for statistical comparison.

### 2.4. Carbohydrate Ligand Binding

Assay: VLPs were serially diluted starting at 8 µg/mL onto saliva or PGM coated plates as described and incubated for 1 h at 37 °C [45]. Binding was detected with anti-VLP rabbit hyperimmune sera, followed by anti-rabbit IgG-HRP, and color developed as above. For VLP binding to carbohydrate in the presence of 1% bile, VLPs were serially diluted from 8 µg/mL and color developed and analyzed as above. 

### 2.5. Bile-Titration Assay

For the bile-titration assay, bovine bile extract, (Sigma-Aldrich, St. Louis, MO, USA) was serially diluted starting at 2% in 1 µg/mL VLP blotto solution onto either A or B saliva coated plates. Bound VLP were detected with anti-VLP rabbit hyperimmune sera, followed by anti-rabbit IgG-HRP, and color developed as above. 

### 2.6. Carbohydrate Ligand Blockade 

Assay: For blockade of ligand binding assays, VLPs were pretreated with serially decreasing concentrations of mAbs for 1 h and added to saliva or PGM coated plates for 1h and VLP bound to carbohydrate ligand detected as described above. One percent bile was added during the antibody pretreatment in select experiments, as noted. Percent control binding was defined as the binding in the presence of antibody pretreatment compared to the binding in the absence of antibody multiplied by 100.

### 2.7. Quantification and Statistical Analysis

Antibody EC_50_ and IC_50_ for EIA and blockade of ligand binding assays were determined from log-transformed normalized data fit with non-linear, dose-response sigmoidal curve fit analysis using GraphPad 8.3.0 [30,46,47]. Antibody binding by EIA less than 3× OD background at 2 µg/mL or blockade assays IC_50_ > 2 µg/mL was scored as negative and assigned a value of 4 µg/mL for statistical analysis. For K_d_ and Bmax values, OD were fit using one-site specific dose response curves of non-linear data in GraphPad. Samples were tested in duplicate in a minimum of two independent experiments.

## 3. Results

**Figure 1:**
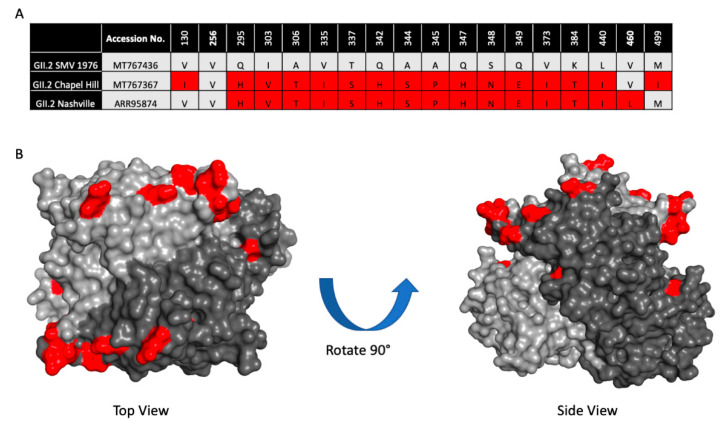
Sequence variation between 2017 GII.2 outbreak strains used in this study and prototype strain GII.2 Snow Mountain Virus (SMV) 1976. (**A**). Capsid amino acid differences between ancestral GII.2 Snow Mountain Virus (GII.2 SMV 1976) and contemporary 2017 GII.2 strains Chapel Hill and Nashville are shown in red. (**B**)**.** Residues in contemporary GII.2 that are different from GII.2 SMV 1976 shown in panel A (red) mapped onto the P domain dimer (light and dark grey).

To determine if the 2016–2017 GII.2 capsid strains had developed residue changes that led to antigenic differences, or an expansion of HBGA binding profiles significant enough to explain the spike in GII.2 outbreaks during the 2016–2017 norovirus season, we first aligned 23 2016–2017 GII.2 capsid sequences collected globally and compared them to the prototype GII.2 strain, GII.2 1976 Snow Mountain Virus (SMV) [40,48,49]. All the GII.2 capsids shared >96% amino acid similarity. For an in-depth analysis, we then selected 2 representative 2017 strains circulating within the United States with complete capsid sequences, GII.2 Chapel Hill, and GII.2 Nashville (Figure 1A). Collectively, the representative strains had >97% amino acid similarity to GII.2 1976, with 18 amino acid residues located within VP1 that differed. RdRP sequence for GII.2 Nashville was found to be GII.P16, reflecting similar RdRP amino acid changes reported in circulating GII.4 2012 strains, while RdRP for GII.2 Chapel Hill was not sequenced [37,41]. The majority of the residue differences clustered around the most surface exposed ridges on the GII.2 capsid (Figure 1B), areas structurally similar to known neutralizing antibody epitopes within the pandemic GII.4 strains [29,30,50]. 

**Figure 2:**
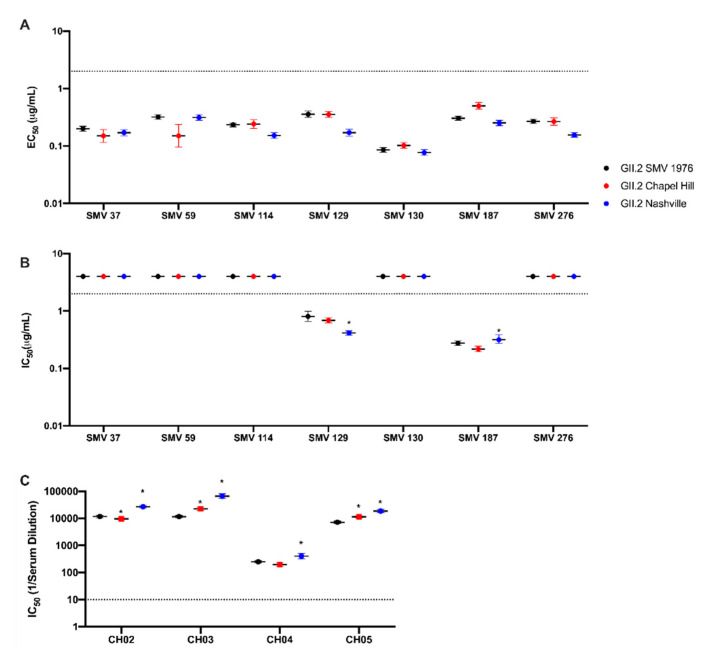
Antibody epitopes are conserved between 2017 GII.2 strains and GII.2 SMV 1976. Contemporary GII.2 Virus Like Particles (VLP) binding to mouse monoclonal antibodies to GII.2 SMV 1976 VLP (Swanstrom et al., 2014). EC_50_ and IC_50_ values were calculated for sigmoidal dose-response curves fit to the percent maximum binding for EIA (**A**), and mean percent control binding for antibody inhibition of ligand binding, blockade assays by mAb (**B**), or human sera from GII.2 infected donors (**C**)**.** Error bars represent 95% confidence intervals. Non-blockade mAbs were assigned an IC_50_ of 4 μg/mL for statistical analysis and are denoted by data markers on the graph above the upper limit of detection (dashed line) for visual comparison. *, VLP with significantly different blockade titer from that of GII.2.SMV 1976 with *p* < 0.05.

VLP of the 2016–2017 GII.2 strains were synthesized (Supplementary Figure S1) and screened for reactivity to a set of monoclonal antibodies derived from mouse hyperimmunization with GII.2 1976 SMV VLP [31]. All monoclonal antibodies to the prototype strain retained binding to contemporary GII.2 strains (Figure 2A). Variation of EC_50_ titers within each antibody for each VLP varied two-fold or less.

Similarly, antibodies SMV129 and SMV187 retained blockade of ligand binding capacity for the contemporary VLP strains (Figure 2B), with respective IC_50_ titers varying up to 2-fold compared to GII.2 1976 SMV. Non-blocking antibodies SMV37, SMV59, SMV114, SMV130, and SMV 276 did not gain blockade capability with the contemporary GII.2 VLPs. Further, sera from GII.2 Chapel Hill infected individuals, CH02-5, were screened against our panel of GII.2 VLP for blockade of ligand binding capacity (Figure 2C) [41]. All four sera demonstrated blockade activity for all VLP, with <6-fold variation observed between each VLP per sera. Thus, available GII.2 monoclonal and polyclonal antibody binding and blockade potency assays did not capture epitope specific changes within the contemporary GII.2 strains or between the contemporary and ancestral GII.2 strains that could explain the spike in GII.2 viruses during the 2016–2017 norovirus season.

**Figure 3:**
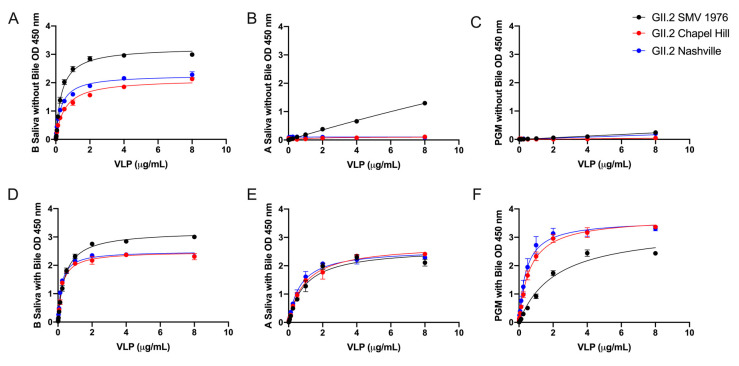
Bile enhances the binding of GII.2 VLP to ligands present in PGM and human type A saliva. VLP were titrated starting against human type B Saliva (**A**,**D**), A Saliva (**B**,**E**), and PGM (**C,F**) with (**D**–**F**) and without (**A**–**C**) 1% bile. OD were fit using one-site specific binding curves, with error bars representing standard error of the mean.

GII.2 1976 SMV VLP preferentially bind to human type B saliva, weakly bind to type A saliva, and do not bind to PGM [31,39,40]. To test if the carbohydrate binding profiles of the contemporary strains were influenced by subtle changes in the capsid sequence, VLPs were serially diluted onto PGM and human type A and B saliva coated plates. All GII.2 VLP bound robustly to B saliva (K_d_ 0.31 to 0.52 µg/mL) (Figure 3A). Neither of the two contemporary strain VLPs bound A saliva or PGM, even at high VLP concentrations (8 µg/mL) (Figure 3B,C and Supplementary Figure S2) indicating that microvariation within the capsid sequence likely modulates ligand binding affinities between similar GII.2 strains, as reported for GII.4 strains [19,41].

To determine if bile could influence the interaction of the GII.2 VLPs with additional secretor ligands, GII.2 VLPs were treated with bile and analyzed for binding to B and A saliva and PGM. An optimal concentration of 1% bile improved the binding of all tested GII.2 VLP to A saliva (Supplementary Figure S3) and moderately improved binding to B saliva, resulting in similar Bmax (1.741–1.780 OD at 450 nm) and K_d_ (0.012–0.025 µg/mL) values for bile enhancement of the contemporary and ancestral strains. Bile restored binding of GII.2 VLP to A saliva with Bmax and K_d_ values within 2-fold of GII.2 binding to B saliva (Figure 3E).

Bile also improved binding to PGM (Figure 3F). As seen for type A saliva, the effect of bile on GII.2 VLP binding was strain dependent, with the greatest improvement on the contemporary GII.2 VLP. GII.2 SMV bound significantly less well to PGM in the presence of bile than the contemporary GII.2 VLP with bile (Figure 3). These data indicate that microvariation within the capsid sequences of GII.2 strains modulates binding to different ligands in the presence of bile and co-factors including bile can significantly influence GII.2 ligand binding affinity.

**Figure 4:**
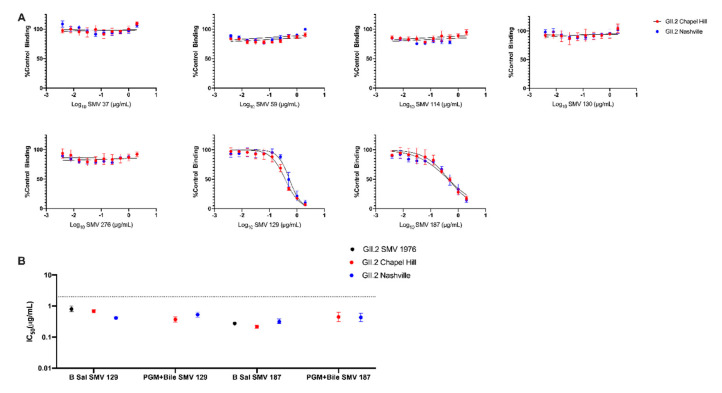
Bile does not affect antibody blockade of Contemporary GII.2 VLP binding to ligands. (**A**) Contemporary GII.2 VLP blockade of PGM binding by mouse monoclonal antibodies is maintained in the presence of 1% bile. Error bars represent standard error of the mean. (**B**) IC_50_ values obtained for SMV 129 and SMV 187 on different ligand sources, with and without bile. Sigmoidal dose–response curves were fit to the mean percent control binding for blockade assays, and the IC_50_ was calculated in μg/mL. Dashed line represents limit of detection. Error represents 95% confidence intervals.

To determine if bile could also impact antibody-VLP interactions, GII.2 blockade mAb were retested for blockade potency using the contemporary GII.2 VLP pre-incubated with both mAb and 1% bile before addition to PGM (Figure 4A). Variation in IC_50_ titer was <2 fold between PGM with bile and B saliva for both SMV 129 and SMV 187 for both contemporary VLP (Figure 4B). mAbs SMV 37, SMV 59, SMV 114, SMV 130, and SMV 276 did not inhibit VLP binding, in agreement with B saliva blockade assays (Figure 4A). SMV did not bind to PGM under any tested condition. These data support bile as an important cofactor for GII.2 HBGA binding and indicate that bile-binding to the capsid did not alter capsid antigenicity.

**Figure 5:**
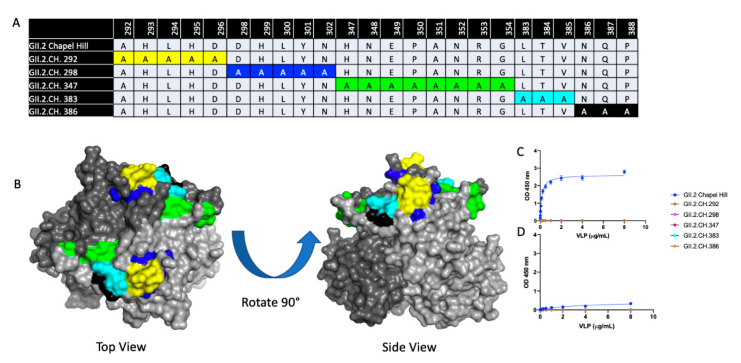
Characterization of GII.2 Chapel Hill Alanine mutant VLPs. (**A**) A panel of 5 alanine mutant VLPs were constructed comprising variable residues between GII.2 1976 SMV and GII.2 Chapel Hill within the P2 domain. Sequence alignments of variable residues within the 5 alanine mutants are compared against the Chapel Hill backbone. Color coordinated highlighted regions are highlighted on the GII.2 P domain dimer in (**B**). VLP binding to A and B saliva (**C**,**D**) were analyzed via one-site specific binding curve, fitted with error bars representing standard error of the mean.

To better understand residues on the GII.2 capsid that were important for mAb and HBGA recognition, a panel of 5 GII.2 Chapel Hill VLPs were designed with alanine mutations in regions where microvariation was observed between the prototypic 1976 and Chapel Hill GII.2 strains. (Figure 5A,B). Particle integrity and carbohydrate binding profiles were screened for each of these mutants, and while the VLP were indistinguishable from the native GII.2 Chapel Hill strain via electron microscopy, all lost carbohydrate binding to both A and B saliva (Figure 5C), supporting previous reports demonstrating that amino acids within the P2 domain are important for HBGA recognition [32,51].

**Figure 6:**
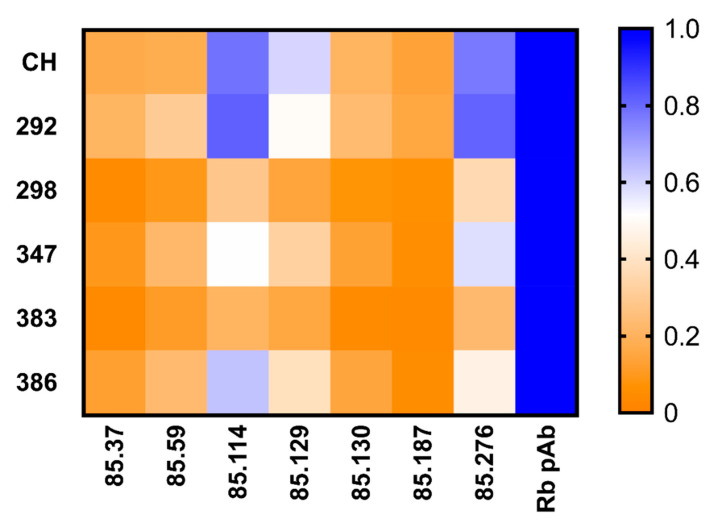
Mutations in surface exposed regions of GII.2 Chapel Hill show variation in mAb recognition: One site specific binding curves were fit to find the maximum binding, Bmax, values of GII.2 derived mouse mAb binding to GII.2 Chapel Hill alanine mutated VLPs, and normalized across VLP to the rabbit polyclonal sera Bmax value with proportion high to low (blue to orange).

The alanine mutant VLPs were then screened by EIA for their reactivity to GII.2 rabbit polyclonal sera and our panel of GII.2 mAbs, and the binding patterns compared to GII.2 Chapel Hill VLP (Figure 6). Respective Bmax and K_d_ values were within <1.5 and <3-fold for the alanine mutant VLPs and wild-type GII.2 Chapel Hill for rabbit polyclonal sera, indicating that subtle variation within the alanine mutant VLPs did not impact particle or global epitope integrity (Supplementary Figure S1). Alanine mutations did not ablate binding of any monoclonal antibody (Figure 6). EC_50_ variation between wild-type GII.2 Chapel Hill and the alanine mutant VLPs was less than one log difference. To evaluate minor changes in antigenicity that could be affecting monoclonal binding, we compared the Bmax values for each mAb to the Bmax for rabbit polyclonal sera for each VLP. All alanine mutated regions decreased binding of the blocking mAbs, SMV 129, and SMV 187, indicating that the blocking epitopes recognized by these mAbs are located within the P2 domain, as reported for GII.4 blocking epitopes [29,30,50]. Alanine mutations in GII.2.CH 292 and GII.2.CH.386 increased binding of SMV 114, and GII.2.CH.292 mutations increased binding to SMV 276, suggesting these residues may sterically hinder binding of these mAbs. Mutations in GII.2CH.298, GII.2.CH.347, and GII.2.CH 383 reduced binding to SMV 114, indicating that residues 298–302 and 383–385 also affect binding of SMV114. Overall, three of the five mutated regions in the P2 domain, GII.2.CH.298, GII.CH.347, and GII.2.CH.383, reduced binding of all tested GII.2 mAb in comparison to GII.2 Chapel Hill, supporting the P2 domain as the major antigenic region of GII.2 HuNoV.

**Figure 7:**
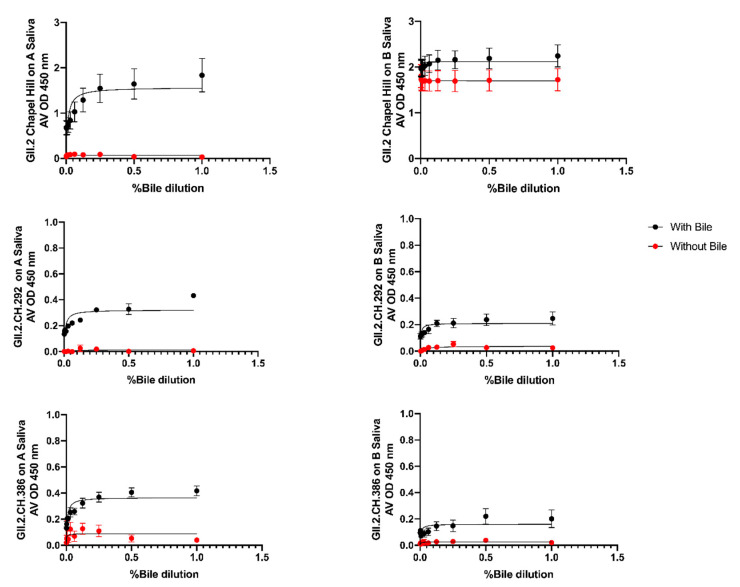
Bile Restores A and B saliva binding in two of the GII.2.CH Alanine mutants: GII.2.CH alanine mutants were screened for binding to A and B saliva in the presence of bile. One-site specific binding curves were fit with error bars representing standard error of the mean.

To assess if bile could recover VLP-HBGA recognition of the alanine mutant VLPs, VLP were treated with bile and evaluated for binding to A and B Saliva. Under standard assay conditions, no binding was detected for any alanine mutant VLP. At 1 µg/mL VLP, GII.2.CH 292 and GII.2.CH 386 gained modest binding to both A and B saliva in the presence of bile (Figure 7). Bile restoration of binding of these VLPs suggests that residue changes within the GII.2.CH 292 and GII.2.CH 386 VLPs were more distal to the carbohydrate binding pocket, and had less of an impact on HBGA recognition as compared to the other 3 alanine mutant VLPs, GII.2.CH 298, GII.2.CH 347, and GII.2.CH 383.

## 4. Discussion

Mechanisms of emergence of GII.2 human noroviruses are not as clearly understood as those of GII.4 pandemic strains, which have been characterized by antigenic drift in known antibody epitopes and recombination events with polymerases from other human norovirus genotypes [17,19,21,27,28,29,30]. Current methodology centers around analyzing antigenic variation of capsids due to the lack of targeted assays to evaluate the effect of variation within polymerase and VP2 sequences. Here, we compared capsid antigenic and ligand binding characteristics of two GII.2 strains circulating during 2016–2017 with the prototypical GII.2 SMV 1976 strain to elucidate potential mechanistic explanations for the surge in GII.2 strains during that time period. Microvariations within the GII.2 capsids did not lead to large changes in polyclonal sera or monoclonal antibody recognition or function. Microvariation within the GII.2 capsids modified ligand binding affinity, with contemporary GII.2 VLPs displaying a reduced affinity to A saliva HBGA in comparison to the prototypic GII.2 VLP. Reduced ligand binding was compensated for by addition of bile, as supported by previous studies [41,52]. While bile may induce a conformational shift favoring ligand-interaction stabilization in GII.2 capsids, the conformational shift did not abrogate binding of any of the mAbs tested, suggesting that bile binding to GII.2 VLP does not significantly modulate antigenicity.

Understanding the gastrointestinal microbiome is critical for studying host ligand interaction with HuNoV. Studies of Porcine Enteric Calicivirus have demonstrated the importance of bile in both viral infectivity and propagation in porcine cell cultures [53]. Bile from porcine, piglet, and human sources has been shown to enhance growth of select HuNoV genotypes within Human Intestinal Enteroid (HIE) cells [43,54,55]. Additionally, improved binding of GII.2 VLP to non-secretor salivary ligands has been demonstrated in the presence of bile [41]. Further, recent molecular interaction studies utilizing GII.1 P domains show that bile acids such as GCDCA and TCA bind to amino acids within a partially conserved hydrophobic pocket of the capsid in GII strains that is distinguished from known HBGA binding sites [52]. GCDCA binding stabilizes the A and B loops located within the HBGA binding pocket of these VLP and directly positions the side chain of Asp375 to stabilize the interaction with the fucose of secretor HBGAs, most notably in non-epidemic/pandemic HuNoV strains [52]. These data suggest that bile is a cofactor for select HuNoV genotypes binding to HBGA ligands and indicate that multiple factors such as host genetics and gastrointestinal microbiome can influence HuNoV susceptibility.

The impact on GII.2 emergence through altered susceptible populations based on the variation in GII.2 HBGA binding profiles characterized here is unknown. Concurrently, no clear antigenic changes were identified between contemporary and ancestral GII.2 strains. Alanine mutation of regions of the capsid support GII.2 blockade epitopes within the P2 domain, as reported for GII.4 HuNoVs [29,30,50]. Loss of HBGA binding of the alanine mutated VLPs limited antigenicity screening to antibody binding. Antibody binding is a less robust metric of antigenic change than antibody blockade of ligand binding. Further studies and larger panels of GII.2 mAbs are needed to map blockade epitopes of GII.2 strains and to inform surveillance parameters, as has been implemented for GII.4 strains, and to define serotypes for vaccine development. Additionally, future research should be directed toward studying capsid plasticity and conformational changes within the GII.2 P domain in the presence of various extracellular factors to examine allosteric effects for antibody recognition, as examined for other HuNoV genotypes and mouse norovirus [30,47,56,57]. 

Previous reports suggest that recombination events between the GII.2 capsid and the P16 polymerase are associated with GII.2 emergence during 2016–2017; however, empirical support for this observation is lacking [35,36]. Given that GII.2 has circulated with versions of the P16 polymerase previously and with other polymerase types during the 2016–2017 surge, recombination is unlikely to be the primary explanation for new strain emergence. The limited antigenic and HBGA binding variation described here across GII.2 strains is also unlikely to be the primary explanation for the 2016–2017 GII.2 emergence. These data together with the observed global surge in GII.17 strains in 2014–2015 indicate that shifts in herd immunity may be the leading catalyst for emergence of GII strains that typically circulate at low levels. Herd immunity shifts could result from both waning over time or reshaping by current strain exposure. Both GII.17 and GII.2 emerged during a period when herd immunity globally has been shifted by extensive GII.4 2012 Sydney exposure. It is possible that the Sydney-focused remodeled immune profile of the population created gaps in immunity that allowed GII.17 and GII.2 strains to infect adults and to be detected in outbreak investigations.

HuNoVs persist in the human population via multiple mechanisms: genetic recombination (many genotypes), evolution in HBGA binding profiles and antibody epitopes (GII.4 genotype), and waning/reshaping of immunity (likely all genotypes) [58]. Future virus propagation studies in HIE cultures should elucidate the impact of recombination on viral fitness. Serum antibody repertoire studies will define antibody immunity before and after infection, characterize the breadth of immunity across HuNoV genotypes, and define the effects of immune reshaping on breadth over time, significantly increasing our understanding of new HuNoV strain emergence.

## Supplementary Materials

The following are available online at https://www.mdpi.com/1999-4915/12/9/989/s1, Figure S1: Negative-stain electron micrographs of GII.2 virus-like particles, Figure S2: GII.2 1976 SMV and GII.2 Chapel Hill Recognition of HBGA, Figure S3: Bile Enhances Binding of GII.2 VLP to HBGA.
