# Supplementary material for: Bile Facilitates Human Norovirus Interactions with Diverse Histoblood Group Antigens, Compensating for Capsid Microvariation Observed in 2016–2017 GII.2 Strains
Source: Viruses. 2020 Sep 5;12(9):989. doi: 10.3390/v12090989 (PMC7552067; doi:10.3390/v12090989)
Supplement: Supplementary file 1 [file viruses-12-00989-s001.pdf]

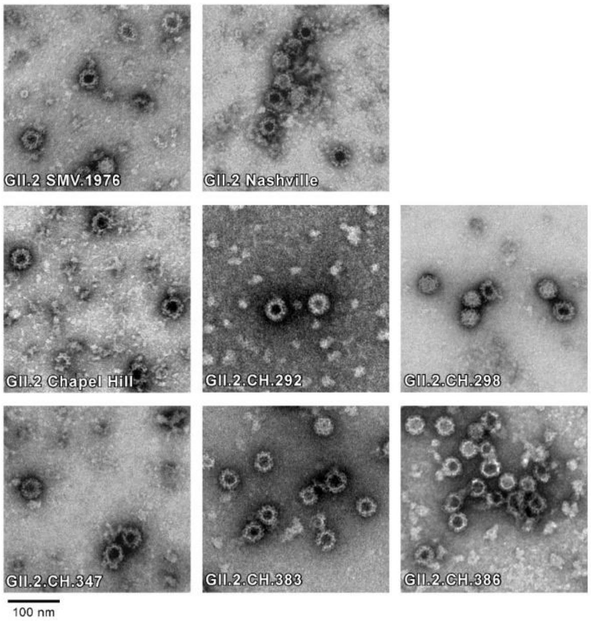

Supplementary Figure S1. Negative-stain electron micrographs of GII.2 virus-like particles.

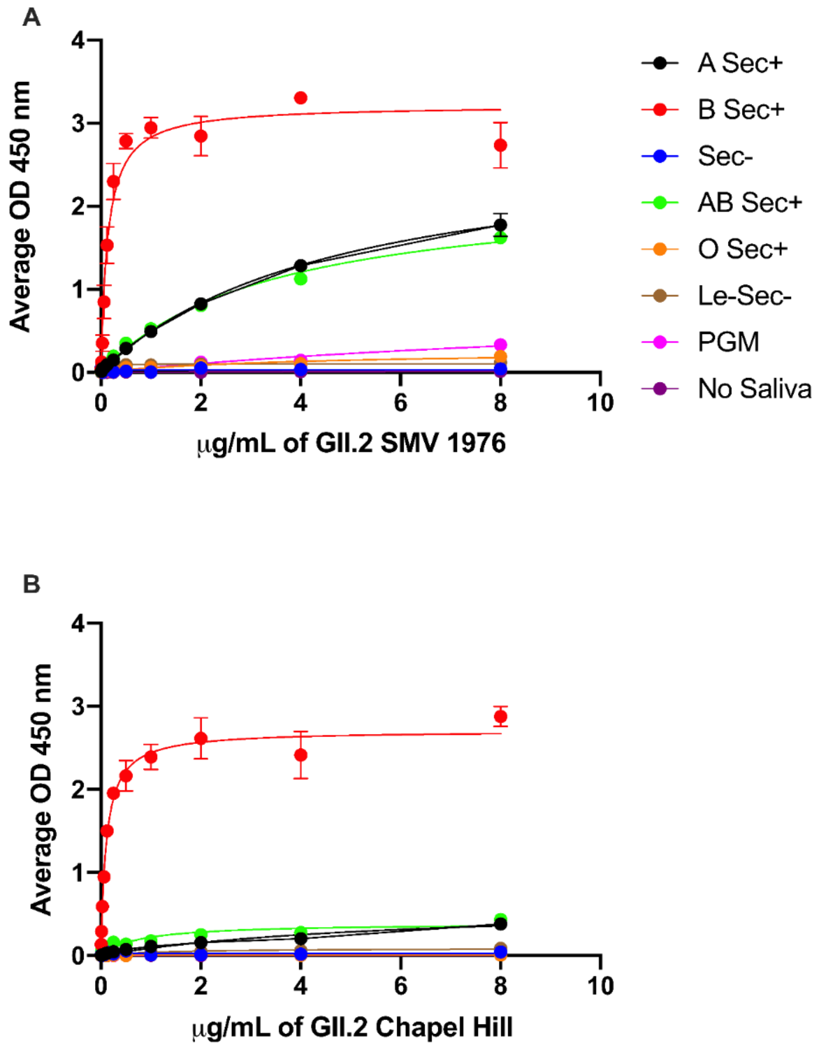

Supplementary Figure S2. GII.2 1976 SMV and GII.2 Chapel Hill Recognition of HBGA**.** GII.2 SMV 1976 (**A**) and GII.2 Chapel Hill (**B**) VLPs were titrated against archived saliva samples of known HBGA expression profile. One-site specific binding curves were fitted with error bars representing the standard error of the mean.

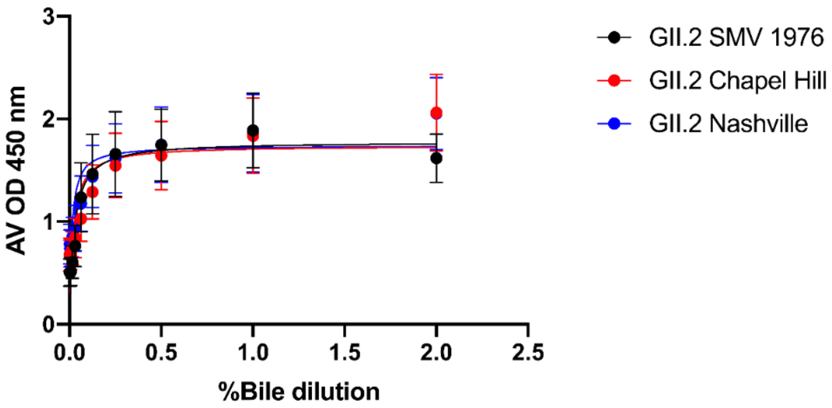

Supplementary Figure S3. Bile Enhances Binding of GII.2 VLP to HBGA.
